# Supplementary material for: Altered Expression of TMEM43 Causes Abnormal Cardiac Structure and Function in Zebrafish
Source: Int J Mol Sci. 2022 Aug 23;23(17):9530. doi: 10.3390/ijms23179530 (PMC9455580; doi:10.3390/ijms23179530)
Supplement: Supplementary file 1 [file ijms-23-09530-s001.zip › ijms-1859884-supplementary.pdf]

Supplementary Materials for

Altered Expression of *TMEM43* Causes Abnormal Cardiac Structure and Function in Zebrafish

Miriam Zink, Anne Seewald, Mareike Rohrbach, Andreas Brodehl, Daniel Liedtke, Tatjana Williams, Sarah J. Childs and Brenda Gerull[1]

[1]Author for correspondence:
Prof. Brenda Gerull
E-mail: Gerull_B@ukw.de
Tel.: +49 931 – 201 46457

This file includes:

Supplementary Figures S1 to S8

Supplementary Table S1

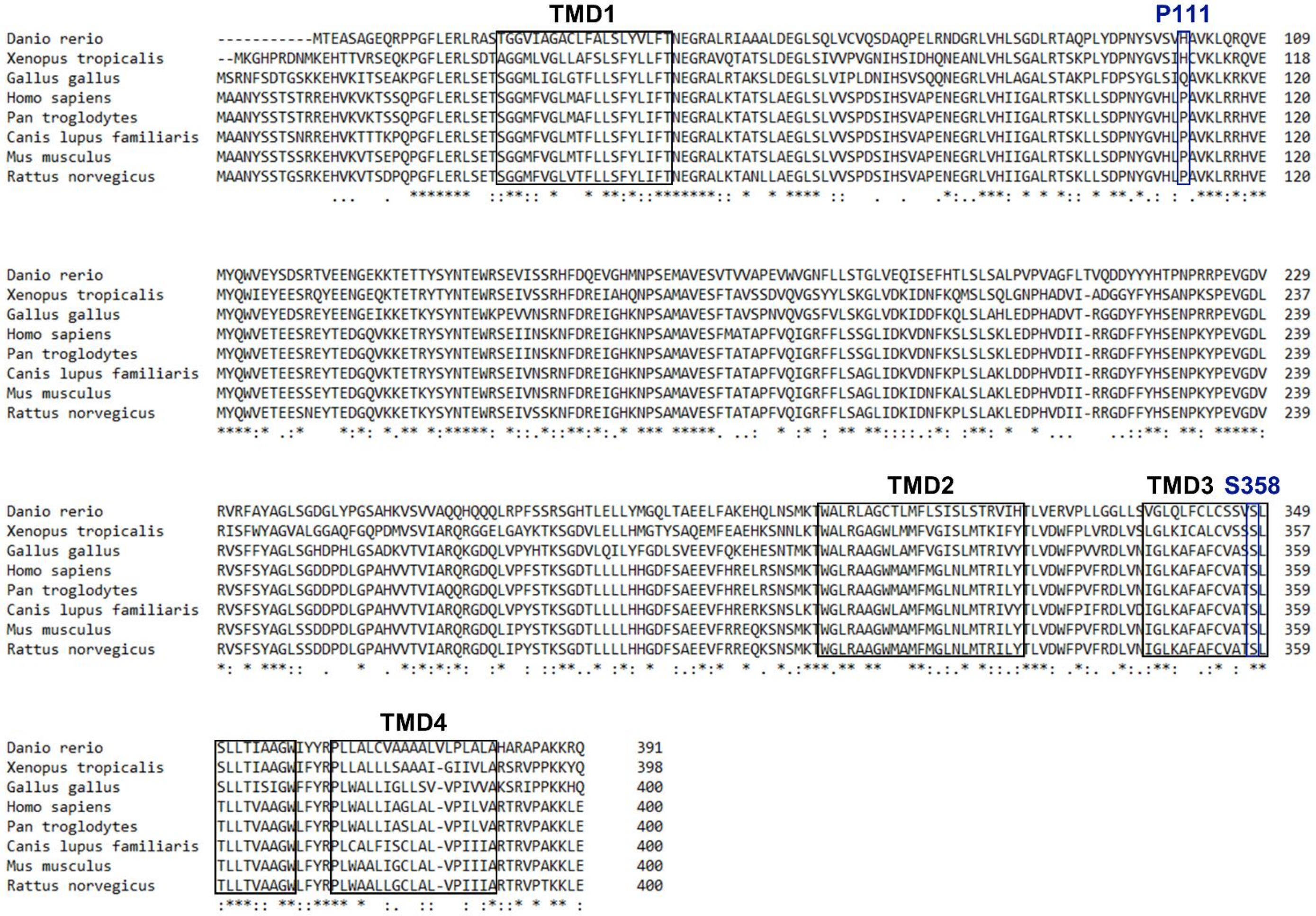

Figure S1: TMEM43 is evolutionary highly conserved.

Multiple amino acid sequence alignment of TMEM43 orthologues across eight eukaryotic species. To identify a *tmem43* zebrafish ortholog, the corresponding amino acid sequences from *Danio rerio* (AAI54205.1) were aligned with *Xenopus tropicalis* (XP_002938277), *Gallus gallus* (XP_414378), *Canis lupus familiaris* (XP_541751), *Homo sapiens* (NP_077310.1), *Pan troglodytes* (XP_001157301), *Mus musculus* (NP_083042.1) and *Rattus norvegicus* (NP_001007746) using the Clustal Omega (1.2.4) program. Fully conserved residues are indicated with an asterisk, highly conserved residues with a colon, and weaker conserved residues are indicated with a dot. The black boxes outline the predicted transmembrane domains (TMD) TMD1-TMD4 and the blue boxes highlight the position of the human TMEM43 variants p.P111L and p.S358L. In zebrafish there is a histidine instead of a proline at amino acid position 111.

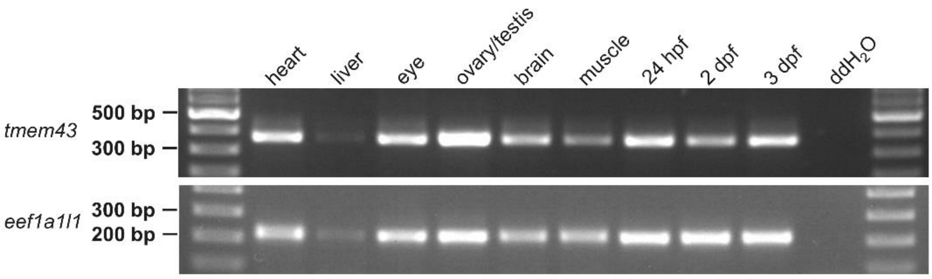

Figure S2: *tmem43* is expressed in a wide range of developmental stages and adult tissues in zebrafish.
RT-PCR analysis of *tmem43* expression in adult tissue and during different embryonic developmental stages.

A

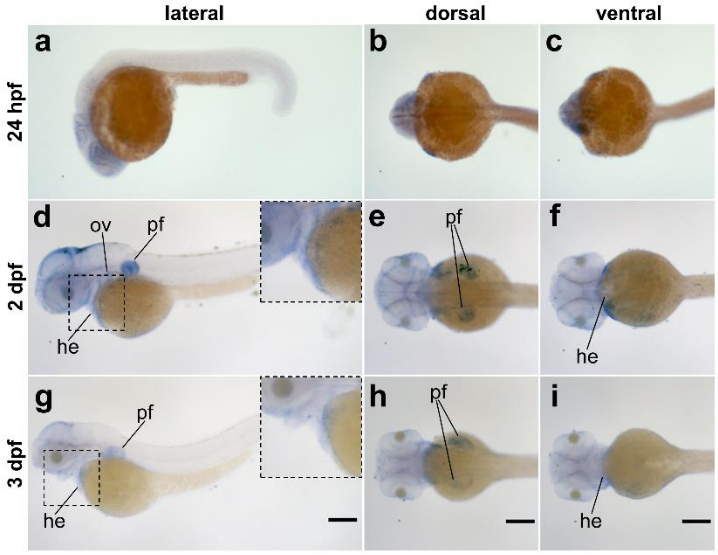

B

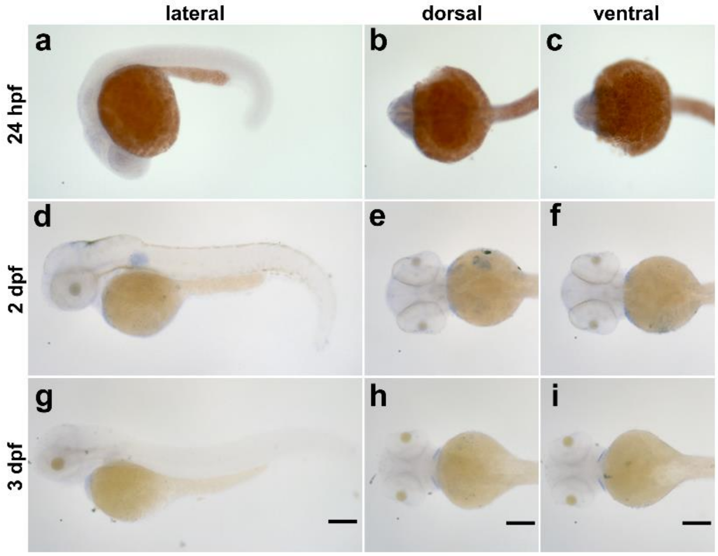

Figure S3: Spatiotemporal expression analysis of zebrafish *tmem43* during embryonic development.

(**A**) Representative images of zebrafish embryos at indicated developmental stages stained by whole-mount *in situ* hybridization (WISH) using a *tmem43* antisense probe. (a – d) Signals in the head region at 24 hours post fertilization (hpf). (d – i) From 2 days post fertilization (dpf) onwards, additional *tmem43* expression appears in brain tissues (tectum, mid-hindbrain-boundary, and cerebellum), the heart (he), the pectoral fin (pf) and the otic vesicle (ov). Insets show higher magnification of the boxed region. (**B**) Sense probe showing absence of *tmem43* signal. (**A**,**B**) Scale bars = 200 µm. WISH experiments were performed in tricplicate, with *n* = 10 individuals per developmental stage. (a, d, g) Lateral views with anterior to the left. (b, e, h) Dorsal views with anterior to the left. (c, f, i) Ventral views with anterior to the left.

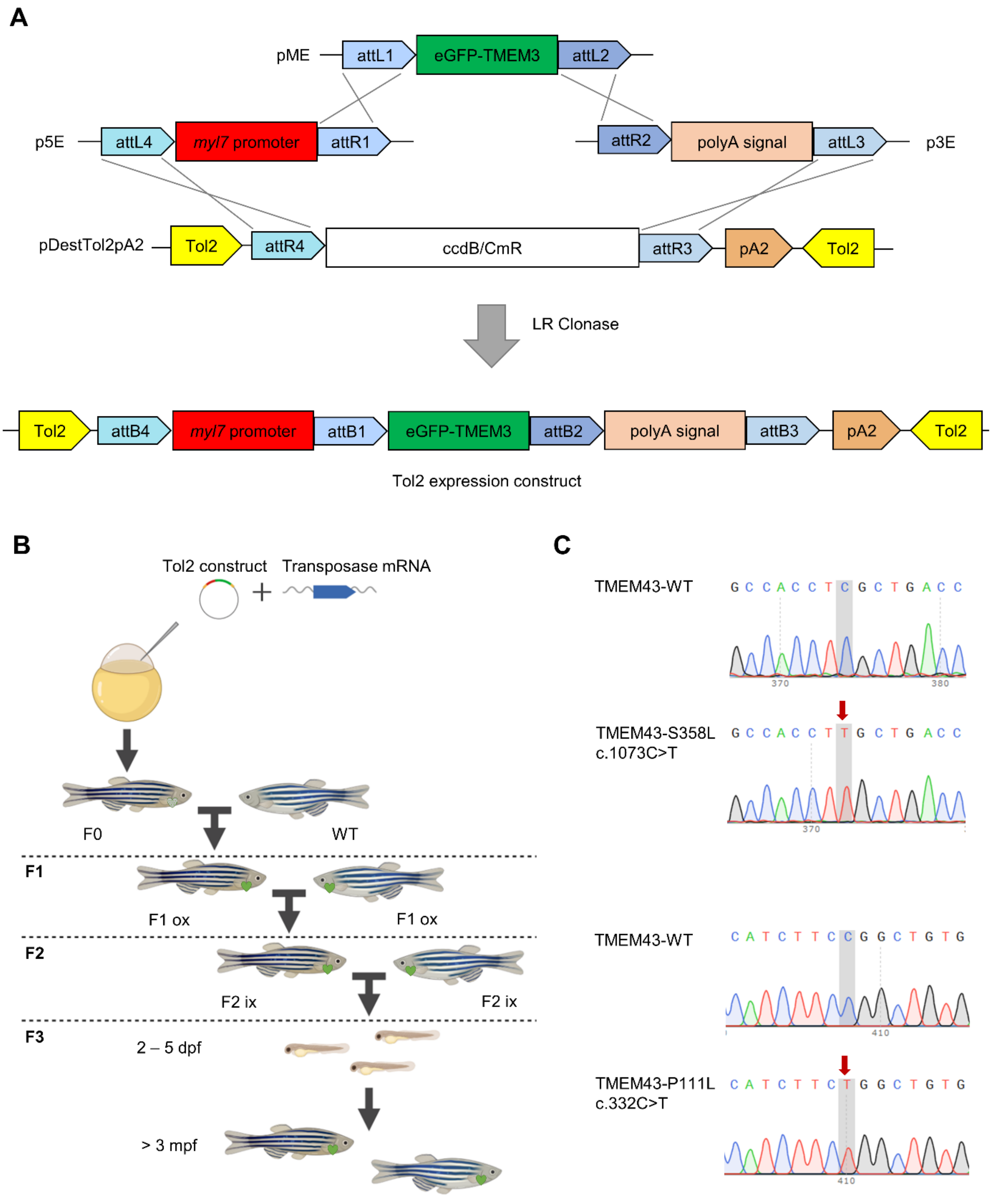

Figure S4: Generation of a transgenic TMEM43 zebrafish model using the Tol2 system.
(**A**) Schematic of the Gateway cloning strategy. To generate the expression construct, a three-way multisite Gateway cloning approach with three entry clones and the pDestTol2pA2 destination vector was performed. The final transposon donor plasmid contains the Tol2 construct with the cardiomyocyte-specific *myl7* promoter, the reportergene *eGFP* fused to the cDNA sequence encoding for full-length human *TMEM43*, either wild-type (WT) or with the respective mutations (c.1073C>T, p.S358L; c.332C>T, p.P111L). (**B**) Transgenesis in zebrafish. A synthetic transposase mRNA and the transposon donor plasmid containing the expression construct represented in (**A**) are co-injected into fertilized eggs in one-cell stage. The construct is stably integrated into the zebrafish genome via the Tol2 isomerase.

Thereby, the position and number of integration events is random. Germline transmission of the transgene can be selected in the F1 generation by outcrossing the injected founder fish with wild-type fish. For all experiments in this manuscript, progeny of F2 in-cross mating were used. (**C**) Validation of construct integration into the zebrafish genome via Sanger sequencing for wild-type, TMEM43-S358L (upper panel) and TMEM43-P111L (lower panel) zebrafish. The positions of the *TMEM43* missense mutations are highlighted in grey, as well as the corresponding position in the wild-type. Red arrows indicate the substituted nucleotide. Created with BioRender.com (accessed on 13 August 2022).

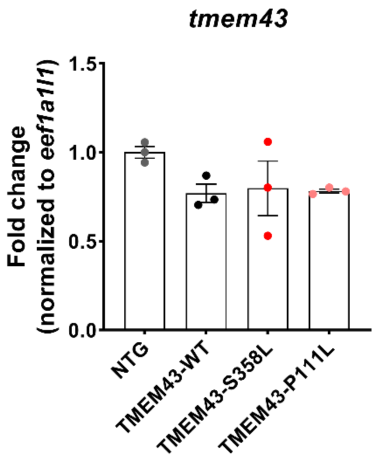

Figure S5: *tmem43* expression in ventricles of transgenic zebrafish.
Quantitative reverse transcription PCR demonstrates transcript reduction of endogenous zebrafish *tmem43* in adult ventricles of all transgenic TMEM43 lines compared to the non-transgenic control (NTG). There is no significant difference in *tmem43* expression between the different transgenic lines. Each replicate consists of a pool of 2-7 ventricles. Relative fold change is normalized to *tmem43* expression in NTG. One-way ANOVA with Bonferroni's multiple comparison test, * $p \leq 0.05$. Error bars correspond to SEM.

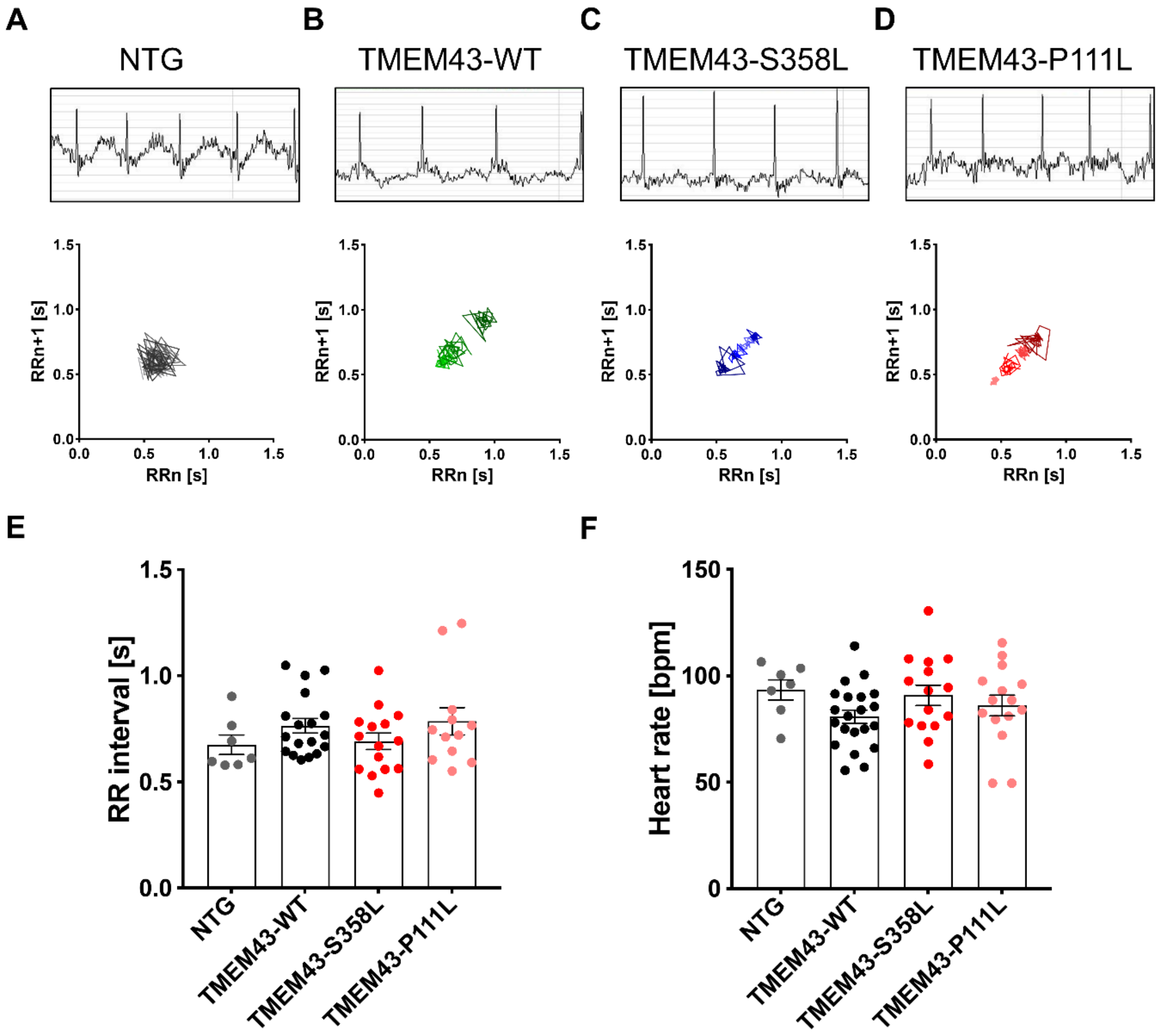

Figure S6: Normal cardiac electrophysiology of 5 mpf adult zebrafish.
Electrical conduction properties of adult hearts were assessed by surface ECG analysis. (**A** – **D**) Representative ECG traces and Poincaré plots of consecutive RR intervals from four individual fish of each genotype. Individual fish are each represented by a different colour shading. (E) Quantification of RR interval shows no significant differences between the analyzed genotypes. Individual data points represent the mean RR interval of each fish. (**F**) Quantification of heart rate of anesthetized zebrafish shows no significant differences between the analyzed genotypes. (**E**,**F**) For all graphs, significance was determined by one-way ANOVA with Bonferroni‘s multiple comparison test, * $p \leq 0.05$. Error bars correspond to SEM.

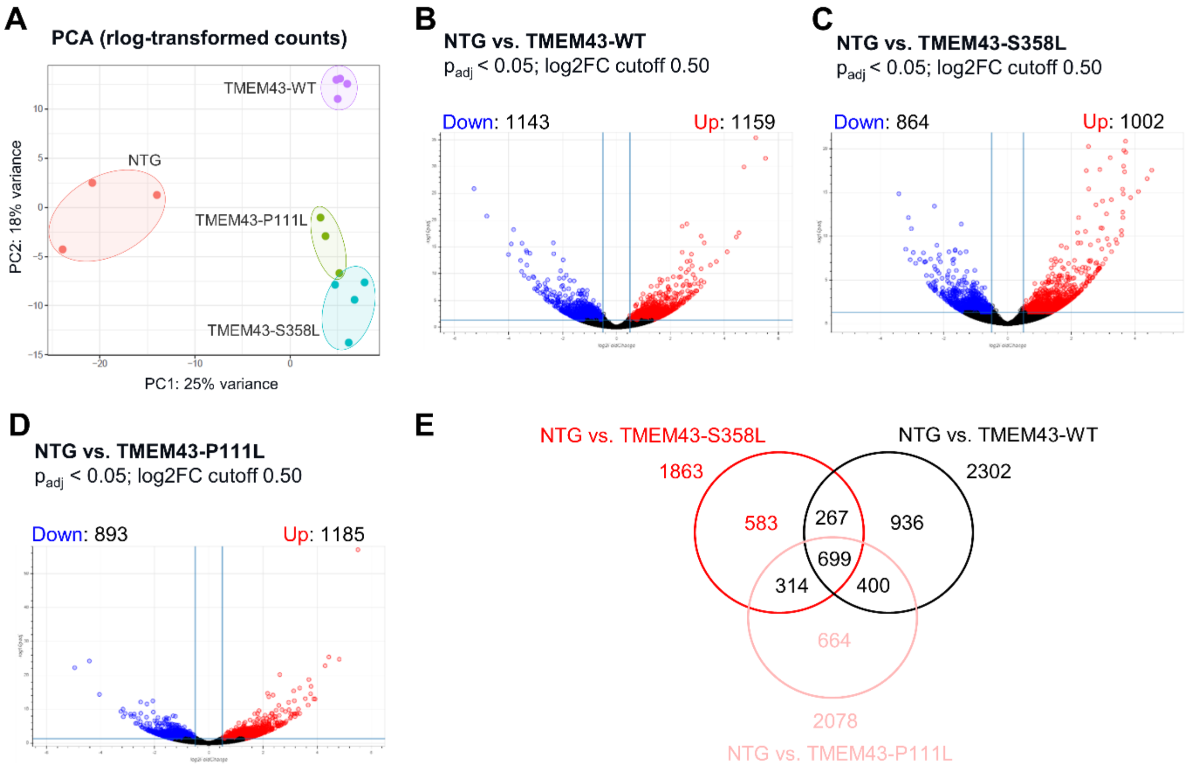

Figure S7: Transcriptome of ventricular tissue in adult TMEM43 zebrafish.
(**A**) Principle component analysis (PCA) showing four clusters of samples, indicating the significant differentiation between the genotypes. (**B** – **C**) Volcano plots showing differentially expressed genes (DEGs) in ventricular tissue isolated from adult zebrafish from (**B**) NTG vs. TMEM43-WT, (**C**) NTG vs. TMEM43-S358L and (**D**) NTG vs. TMEM43-P111L. The horizontal blue line indicates significance level at $p_{adj} < 0.05$, the vertical blue lines indicate log2 fold change (log2fc) cut-off at 0.5. Blue indicate down-regulated, red up-regulated, and black unchanged transcripts. $n \geq 3$; each replicate consists of a pool of 2-7 ventricles. (**E**) Venn diagram showing comparison of the DEGs between NTG and transgenic lines, identifying DEGs common to two or all three genotypes or present exclusively in one.

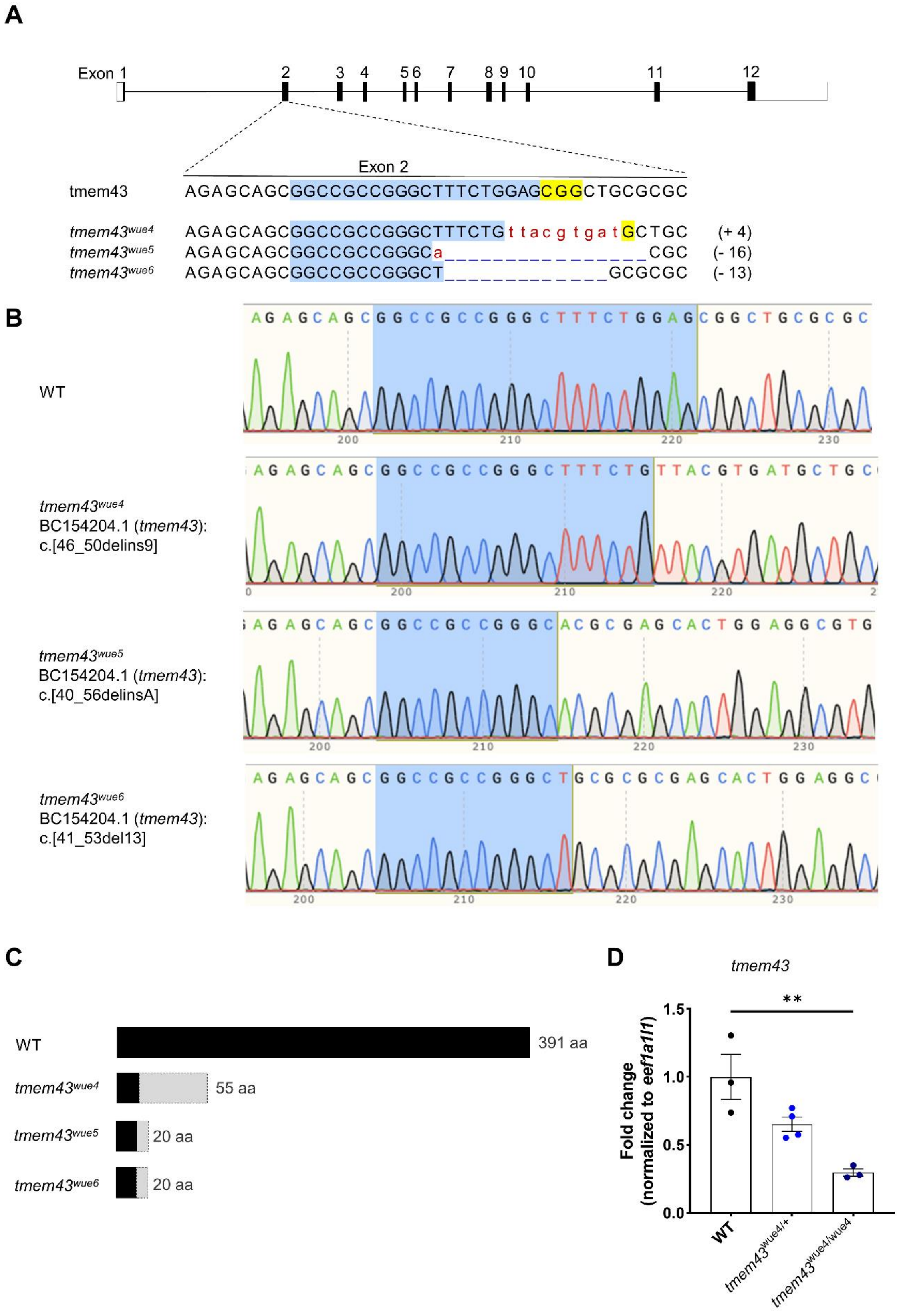
A
Exon 1
2
3
4
5 6
7
8 9 10
11
12
Exon 2
tmem43
AGAGCAGCGGCCGCCGGGCTTTCTGGAGCGGCTGCGCGC
tmem43wue4
AGAGCAGCGGCCGCCGGGCTTTCTGttacgtgatGCTGC
(+ 4)
tmem43wue5
AGAGCAGCGGCCGCCGGGCa_______________CGC
(- 16)
tmem43wue6
AGAGCAGCGGCCGCCGGGCT_____________GCGCGC
(- 13)
B
WT
tmem43wue4
BC154204.1 (tmem43):
c.[46_50delins9]
tmem43wue5
BC154204.1 (tmem43):
c.[40_56delinsA]
tmem43wue6
BC154204.1 (tmem43):
c.[41_53del13]
200
210
220
230
C
WT
391 aa
tmem43wue4
55 aa
tmem43wue5
20 aa
tmem43wue6
20 aa
D
tmem43
**
Fold change
(normalized to eef1a1l1)
0.0
0.5
1.0
1.5
WT
tmem43wue4/+
tmem43wue4/wue4

Figure S8: Generation of *tmem43* mutant zebrafish using CRISPR/Cas9.
(**A**) Schematic illustration showing the genomic wild-type (WT) reference sequence in exon 2 of *tmem43* targeted for the induction of knock-out mutations. The target sequence is highlighted in blue and the PAM sequence is highlighted in yellow. Inserted nucleotides are highlighted by red lowercase letters and deletions are highlighted by blue dashes. The net change of each indel mutation is noted in brackets at the right end of each sequence. (**B**) Sanger sequencing traces for WT and homozygous zebrafish lines $tmem43^{wue4}$, $tmem43^{wue5}$ *and* $tmem43^{wue6}$. The target sequence is highlighted in blue. (**C**) Protein frame of WT Tmem43 and the predicted truncated proteins resulting from the frameshift caused by the indel mutations. In $tmem43^{wue4}$ the first 15 amino acids (aa; black) are identical to those of the WT Tmem43 protein, followed by 40 altered amino acids (grey) before termination. In $tmem43^{wue5}$ the first 13 aa (black) are identical to those of the WT protein, followed by 7 altered amino acids (grey) before termination. The 13 bp deletion in $tmem43^{wue6}$ results in a truncated protein of 20 aa with the first 13 aa (black) identical of those of the WT Tmem43 protein, followed by 7 nonsense amino acids (grey). (**D**) Quantitative reverse transcription PCR demonstrates transcript reduction of *tmem43* in skeletal muscle of both *tmem43* heterozygous ($tmem43^{wue4/+}$) and homozygous ($tmem43^{wue4/wue4}$) mutants. Relative *tmem43* expression is calculated in relation to *tmem43* expression levels in WT fish. Significance was determined by one-way ANOVA with Bonferroni‘s multiple comparison test, ** $p \leq 0.01$. Error bars correspond to SEM.

Table S1: Predicted effect of different TMEM43-p.P111 variants

	gnomAD v2.2.1	BayesDel-addAF		BayesDel-noAF		PON-P2		ClinPred		PolyPhen-2	
	**Allele frequency**	**Score**	**Prediction**	**Score**	**Prediction**	**Score**	**Prediction**	**Score**	**Prediction**	**Score**	**Prediction**
**c.331C>G (p.P111A)**	-	-0.110083	benign	-0.395903	benign	0.174	benign	0.605761408805847	likely pathogenic	0.304	benign
**c.332C>A (p.P111Q)**	-	-0.189698	benign	-0.510265	benign	0.169	benign	0.495748847723007	benign	0.015	benign
**c.332C>T (p.P111L)**	0.000007959	-0.16219	benign	-0.373897	benign	0.206	benign	0.353343665599823	benign	0.749	possibly damaging

The bioinformatic algorithms of BayesDel-addAF, BayesDel-noAF, PON-P2, ClinPred and PolyPhen-2 were used to predict the effect of the different missense TMEM43 variants at amino acid position p.P111.
